# The role of long non-coding RNAs in therapy resistance of cervical cancer and therapeutic potential

**DOI:** 10.3389/fcell.2026.1746979

**Published:** 2026-04-08

**Authors:** Zhenglin He, Hanming Hao, Xuechao Li, Yishuo Ji, Kai Zhao, Lingkai Zhang, Yi Jin, Xue Wang

**Affiliations:** 1 China-Japan Union Hospital of Jilin University, Jilin University, Changchun, China; 2 College of Basic Medical Sciences, Jilin University, Changchun, China; 3 School and Hospital of Stomatology, Jilin University, Changchun, China; 4 The First Hospital of Jilin University, Jilin University, Changchun, China; 5 Department of Immunobiology, Yale University School of Medicine, New Haven, CT, United States; 6 Center of Molecular and Cellular Oncology, Yale Cancer Center, Yale University, New Haven, CT, United States; 7 State Key Laboratory of Female Fertility Promotion, Center for Reproductive Medicine, Department of Obstetrics and Gynecology, Peking University Third Hospital, Beijing, China; 8 School of Cellular and Molecular Medicine, University of Bristol, Bristol, United Kingdom

**Keywords:** biomarker, cervical cancer, long non-coding RNAs, therapeutic target, therapy resistance

## Abstract

Despite significant advances in preventive and early-detection strategies, cervical cancer (CC) continues to pose a substantial clinical burden, especially when confronting the challenge of therapy-resistant or recurrent disease. Long non-coding RNAs (lncRNAs) have emerged as crucial regulators in the development and progression of CC, particularly in mediating resistance to conventional and emerging therapies, including chemotherapy, radiotherapy, and immunotherapy. This review summarizes how specific lncRNAs drive therapeutic resistance through diverse mechanisms such as acting as competitive endogenous RNAs (ceRNAs) to sponge tumor-suppressive microRNAs, and by modulating key signaling pathways like PI3K/Akt and Wnt/β-catenin. Furthermore, we discuss the significant potential of lncRNAs as non-invasive diagnostic and prognostic biomarkers, detectable in liquid biopsies from patient serum or plasma, and as novel therapeutic targets. Advances in targeted strategies have primarily focused on the implementation of precise degradation or interference through antisense oligonucleotides (ASOs) and CRISPR-based systems, highlighting the translational potential of lncRNAs in overcoming refractory therapeutic resistance. Collectively, this review aims to provide a comprehensive overview of the multifaceted roles of lncRNAs in CC therapeutic resistance, offering critical insights that may accelerate the translation of lncRNA-based strategies into clinically actionable interventions to improve patient outcomes in therapy-resistant CC.

## 1 Introduction

Cervical cancer (CC) remains a significant cause of cancer-related morbidity and mortality among women worldwide (Bray et al., 2024). As per data from the 2022 World Cancer Report (GLOBOCAN), it ranks as the fourth most common cancer in women, with an estimated 660,000 new cases and over 350,000 deaths annually, placing a disproportionately high burden on developing regions due to limited access to preventative healthcare (Bray et al., 2024; Duska et al., 2024). While the establishment of screening programs and prophylactic vaccination against high-risk human papillomavirus (hrHPV) has improved prevention, managing advanced or recurrent disease is still a major clinical challenge, as these cases are often characterized by aggressive tumor behavior and limited therapeutic options (Olusola et al., 2019; Moody and Laimins, 2010). The standard treatment for these patients often involves platinum-based chemotherapy, radiotherapy, and increasingly, immunotherapy. However, these standard regimens often achieve limited efficacy in advanced settings, with their effectiveness being severely compromised by the frequent development of therapeutic resistance, which remains a primary cause of treatment failure and poor survival outcomes (Rose, 2003; Fleming, 2023).

The emergence of therapy resistance in CC is a multifactorial process. Key mechanisms include enhanced drug efflux through ATP-binding cassette (ABC) transporters, increased DNA damage repair capacity, evasion of apoptosis, and epithelial-to-mesenchymal transition (EMT) (Zhou M. et al., 2022; Eptaminitaki et al., 2022). Furthermore, the tumor microenvironment (TME), particularly cancer-associated fibroblasts (CAFs) and tumor-infiltrating immune cells, contributes to a protective niche that diminishes treatment efficacy, by secreting pro-survival factors and inducing an immunosuppressive state (Barnestein et al., 2022). Notably, the persistent expression of hrHPV E6 and E7 oncoproteins directly promotes therapy resistance by disrupting tumor suppressor pathways and fostering genomic instability (Molina et al., 2020). Overcoming these intricate mechanisms is crucial for improving patient prognosis. In this complex landscape, dysregulated gene expression, driven not only by genetic mutations but also by profound epigenetic alterations, is a hallmark of cancer progression and therapy resistance (Baldassarre et al., 2025). These epigenetic mechanisms, including DNA methylation, histone modifications, and chromatin remodeling, work in concert with a diverse class of regulatory molecules known as non-coding RNAs (ncRNAs) (Ba et al., 2025).

Among ncRNAs, lncRNAs have emerged as important regulators of gene expression at multiple levels, including the epigenetic level, and as key contributors to carcinogenesis and therapy resistance (He et al., 2020). In CC, specific lncRNAs are dysregulated and have been implicated in promoting malignant phenotypes, including unchecked proliferation, metastatic dissemination, and stemness. Their role in therapeutic resistance is particularly pivotal. They can function as molecular sponges for microRNAs (miRNAs), modulate key signaling pathways, and interact with cellular proteins to enhance cell survival under treatment stress (Yang and Al-Hendy, 2022). Beyond their functional roles, the stability of lncRNAs in bodily fluids makes them promising non-invasive biomarkers for diagnosis and monitoring treatment response (Begliarzade et al., 2023). Moreover, the development of novel targeting strategies, such as RNA interference (RNAi), antisense oligonucleotides (ASOs), the CRISPR/Cas9 system, and exosome-mediated delivery, highlights the potential of lncRNAs as actionable therapeutic targets to circumvent resistance (He et al., 2020).

This review aims to synthesize current knowledge on the mechanisms by which lncRNAs drive resistance to chemotherapy, radiotherapy, and immunotherapy in CC. It will also evaluate their potential as clinical biomarkers and discuss emerging therapeutic strategies targeting lncRNAs to reverse resistance and improve patient outcomes, thereby paving the way for more personalized treatment regimens.

## 2 Regulatory roles of lncRNAs in epigenetic alterations

The central dogma of molecular biology long emphasized protein-coding genes as the primary executors of cellular function. However, the advent of high-throughput sequencing technologies revealed that the vast majority of the human genome is transcribed into non-coding RNAs (ncRNAs), which lack protein-coding potential but are increasingly recognized as critical regulators in both physiological and pathological states (Wang J. et al., 2019). ncRNAs are broadly categorized by size into small non-coding RNAs (e.g., miRNAs, piRNAs, snoRNAs, snRNAs) and lncRNAs, which are defined as transcripts longer than 200 nucleotides (Bridges et al., 2021). Initially considered transcriptional noise, advances in sequencing technologies have illuminated the complex composition of the human transcriptome and the functional significance of lncRNAs (Taniue and Akimitsu, 2021). These molecules exhibit precise spatial and temporal expression patterns and participate in fundamental cellular mechanisms, with their dysregulation being implicated in various diseases, including cancer (Nojima and Proudfoot, 2022). The aberrant expression of lncRNAs can disrupt key cellular processes such as proliferation, apoptosis, and metastasis, thereby playing a fundamental role in cancer initiation and progression (Sanchez Calle et al., 2018).

**FIGURE 1:**
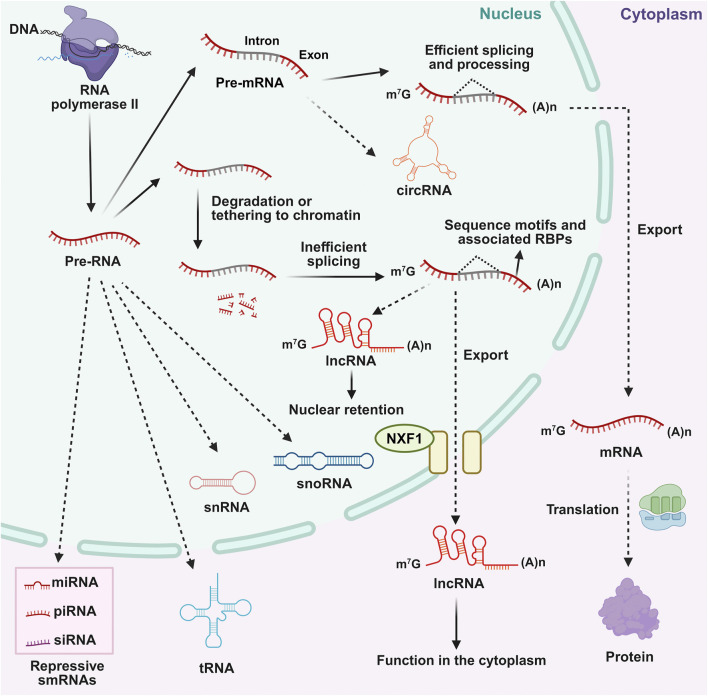
Diverse biogenesis pathway and features of lncRNAs. RNAs are transcribed by RNA polymerase II. lncRNAs can be retained in the nucleus via NXF1-mediated pathways or exported to the cytoplasm to execute functions, distinct from the efficient splicing and translation of canonical mRNAs. Other non-coding RNAs (e.g., circRNAs, snRNAs, tRNAs, and small regulatory RNAs) are also depicted.

**FIGURE 2:**
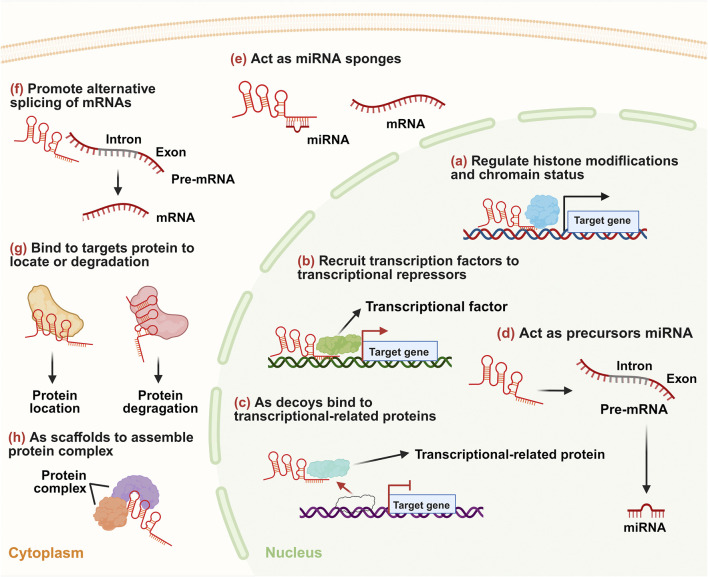
Schematic illustration of the multifaceted regulatory mechanisms of lncRNAs in biological processes. **(a)** lncRNAs exert functions through regulating histone modifications and chromatin status to modulate target gene transcription in the nucleus. **(b)** lncRNAs recruit transcription factors to influence target gene expression. **(c)** lncRNAs bind transcriptional-related proteins, thereby interfering with their regulatory roles. **(d)** lncRNAs act as precursors for miRNA biogenesis. **(e)** lncRNAs serve as miRNA sponges in the cytoplasm to sequester miRNAs and attenuate their post-transcriptional repression on mRNAs. **(f)** lncRNAs promote alternative splicing of mRNAs to generate distinct mRNA isoforms. **(g)** lncRNAs bind to target proteins to regulate their subcellular localization or induce degradation. **(h)** lncRNAs function as scaffolds to assemble protein complexes.

lncRNAs are primarily transcribed by RNA polymerase II as precursors that undergo diverse processing pathways (Figure 1) (Chen and Kim, 2024). Most lncRNAs are capped, spliced, and polyadenylated, then exported to the cytoplasm via NXF1 for functions such as translation modulation or protein/miRNA decoy (Guo et al., 2020). However, lncRNAs are often generated via inefficient splicing, leading to retention of introns or alternative splicing patterns distinct from canonical mRNA processing (Bridges et al., 2021). Despite being exported to the cytoplasm, some mature lncRNAs are retained in the nucleus, where they interact with chromatin or regulatory proteins (Herman et al., 2022). This localization determines their functional roles in gene regulation, chromatin modification, and post-transcriptional control. lncRNAs exert their regulatory functions through sophisticated interactions with DNA, RNA, and proteins, operating at transcriptional, post-transcriptional, and epigenetic levels (Figure 2; Table 1) (Herman et al., 2022). A primary mechanism involves the epigenetic regulation of gene expression. lncRNAs can guide histone-modifying complexes to specific genomic loci, thereby altering chromatin states and influencing the transcriptional activity of neighboring or distant genes (Roberts et al., 2014). For instance, certain lncRNAs recruit repressive complexes that catalyze histone methylation, leading to gene silencing (Peng et al., 2020). In cancer, this mechanism can result in the epigenetic silencing of tumor suppressor genes, providing a growth advantage to cancer cells (Zhu et al., 2020). Conversely, other lncRNAs can promote a transcriptionally active chromatin environment (Ferrer and Dimitrova, 2024). Beyond histone modification, lncRNAs also function as molecular decoys by sequestering transcription factors or other regulatory proteins, preventing them from binding to their DNA targets and thus modulating gene expression programs (Ferrer and Dimitrova, 2024).

**TABLE 1:** Regulatory mechanisms of lncRNAs.

Target	Mechanism	Ref.
DNA level	lncRNAs regulate gene expression by recruiting histone modifiers to specific loci or acting as decoys for transcription factors	Roberts et al. (2014), Ferrer and Dimitrova (2024)
RNA level	lncRNAs act as ceRNAs, sponging miRNAs to prevent mRNA repression. Dysregulation disrupts post-transcriptional control, driving tumorigenesis	Singh et al. (2022)
Protein level	lncRNAs scaffold multi-protein complexes to coordinate signaling, with aberrant function driving oncogenic pathway activation in cancer	Yoon et al. (2013)

At the post-transcriptional level, lncRNAs play pivotal roles as competing endogenous RNAs (ceRNAs) (Singh et al., 2022). They can act as molecular sponges for microRNAs, binding to them and preventing miRNA-mediated repression of target messenger RNAs (mRNAs) (Xue et al., 2022). This ceRNA network represents a critical layer of post-transcriptional control. Dysregulation of this ceRNA network can lead to the overexpression of oncogenes or downregulation of tumor suppressors, thereby driving tumorigenesis (Wang M. et al., 2024). Furthermore, some lncRNAs serve as precursors for the biogenesis of smaller regulatory RNAs (Cheetham et al., 2013). Others influence mRNA stability, alternative splicing patterns, and even the subcellular localization of proteins (Romero-Barrios et al., 2018). Additionally, lncRNAs can function as dynamic scaffolds that facilitate the assembly of multi-protein complexes, bringing together different enzymatic components to coordinate specific cellular responses (Yoon et al., 2013). In the context of cancer, these scaffold functions can aberrantly activate oncogenic signaling pathways, such as those promoting cell survival, invasion, and treatment resistance (Chen et al., 2022). Through these multifaceted mechanisms, lncRNAs establish themselves as integral components of the regulatory circuitry governing gene expression and epigenetic landscapes, with their functional disruption being a hallmark of many cancers, such as CC.

## 3 lncRNAs affecting therapy resistance of CC

**FIGURE 3:**
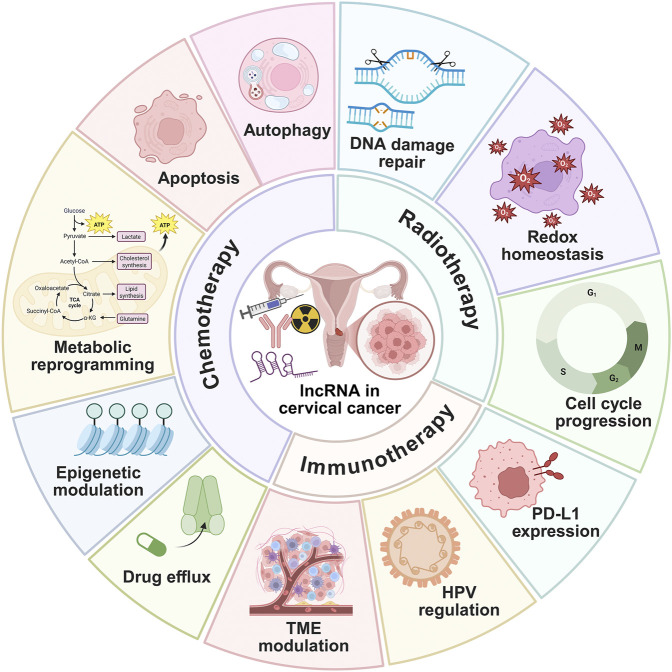
Multifaceted roles of lncRNAs in cervical cancer therapy resistance, including chemo-, radio-, and immuno-therapy resistance.

Treatment resistance remains a major barrier in CC management. Emerging evidence highlights lncRNAs as key regulators of resistance, with distinct roles in chemotherapy, radiotherapy, and immunotherapy (Figure 3). In the following sections, we describe how lncRNAs govern therapeutic resistance in CC (Tables 2, 3).

**TABLE 2:** Upregulated lncRNAs modulating the therapy resistance of cervical cancer.

Function	Medicine	lncRNA	Target	Mechanism	Ref.
Chemo-resistance	Cisplatin	Linc00958	miR-185–5p	Downregulates miR-185–5p to promote RSF-1, increasing cisplatin resistance and angiogenesis via AKT1/GSK3β/VEGFA pathway	Tian et al. (2022a)
		SOX21-AS1	miR-9-3p	Downregulates miR-9-3p to inhibit apoptosis, promoting cisplatin resistance and invasion in cancer	Tian et al. (2022b)
		LOC644656	ZNF143	Co-regulates ZNF143 to promote E6-AP-mediated p53 degradation, enhancing cisplatin-resistant cancer cell growth	Li et al. (2024)
		BCYRN1	miR-330–5p	Downregulates miR-330–5p to promote HMGB3, increasing cisplatin resistance in cancer cells	Sun et al. (2022)
		UCA1	miR-195–5p	Inhibits apoptosis-related proteins to promote cell survival, enhancing cisplatin resistance in cancer cells	Wang et al. (2017)
				Downregulates miR-195–5p to promote IKBKB, increasing cisplatin resistance and malignancy	Wang X et al. (2025)
			Caspase 3, CDK2, p21	Downregulates caspase 3 and upregulates CDK2/survivin, promoting cisplatin resistance and cell proliferation	Wang et al. (2017)
		lnc-DANCR	miR‐665	Downregulates miR-665 to promote cancer progression through molecular sponge function	Cao et al. (2019)
		MIR155HG	miR-409–3p	Downregulates miR-409–3p to promote ZEB1, enhancing EMT and reducing cisplatin sensitivity	Chen et al. (2025a)
		LINC00460	miR-338–3p	Downregulates miR-338–3p to promote TGFBI, promoting cisplatin resistance in cancer	Tian et al. (2024)
		NCK1-AS1	miR-134–5p	Downregulates miR-134–5p to promote MSH2, enhancing cisplatin resistance and inhibiting cell apoptosis	Zhang et al. (2019a)
		NNT-AS1	miR-186	Downregulates miR-186, promoting cisplatin resistance by upregulating HMGB1 and EMT.	Liu et al. (2020)
		OTUD6B-AS1	miR-206	Downregulates miR-206, promoting cisplatin resistance by upregulating CCND2 expression	Hou et al. (2021)
		ZFAS1	-	Promotes cell proliferation/invasion and reduces cisplatin sensitivity	Feng et al. (2019a)
		PCAT6	miR-543	Downregulates miR-543, promoting cisplatin resistance and tumor growth via upregulating ZEB1	Ma et al. (2020)
		ANXA2P2	miR-361–3p	Downregulates miR-361–3p, promoting cisplatin resistance by upregulating SOX9 in a regulatory loop	He et al. (2021a)
	Cisplatin, paclitaxel and docetaxel	HOTAIR	miR-29b	Downregulates miR-29b, promoting EMT and chemo-resistance via PTEN/PI3K axis	Zhang W. et al. (2022)
	Gemcitabine	AFAP1-AS1	miR-7-5p	Downregulates miR-7-5p to promote EGFR, enhancing cancer progression and gemcitabine resistance	Wang M et al. (2024)
		DLG1-AS1	miR-16–5p	Downregulates miR-16–5p to promote HDGF, enhancing cancer resistance to gemcitabine	Zou et al. (2022)
	Paclitaxel	LINC00511	-	Promotes cancer progression, enhancing paclitaxel resistance by regulating cell viability and apoptosis	Mao et al. (2019)
		PVT1	miR-195	Downregulates miR-195 to promote EMT, reducing cancer cell sensitivity to paclitaxel	Shen et al. (2017)
	5-Fu	NEAT1	miR-34a	Downregulates miR-34a to promote LDHA, increasing 5-Fu resistance by enhancing cellular glycolysis	Shao et al. (2021)
Radio-resistance	-	LINC00662	miR-497–5p	Downregulates miR-497–5p to promote CDC25A, enhancing cancer cell proliferation, invasion and radio-resistance	Wei et al. (2020)
	-	LINP1	NHEJ proteins (Ku80 and DNA-PKcs)	Associates with NHEJ pathway proteins to promote DNA repair, increasing radiation resistance	Wang et al. (2018a)
	-	MALAT1	miR-145	Downregulates miR-145, promoting radio-resistance by increasing colony formation and reducing apoptosis	Lu et al. (2016)
	-	HOTAIR	p21	Inhibits p21, promoting radio-resistance, cell proliferation and reducing apoptosis	Jing et al. (2015)
				Upregulates HIF-1α, abrogating radiation-induced cell viability reduction and apoptosis	Li et al. (2018)
Target therapy resistance	-		Wnt/β-Catenin pathway	Interacts with β-catenin to promote Wnt pathway activation, maintaining cancer cell survival despite ICRT14 treatment	Trujano-Camacho et al. (2021)
Immuno-resistance	Ferroptosis	TMPO-AS1	LCN2	Upregulates LCN2 to inhibit ferroptosis, promoting CESC proliferation, migration and tumor growth	Ju et al. (2025)

Abbreviations: RSF-1: Remodeling and spacing factor 1; AKT1: AKT serine/threonine kinase 1; GSK3β: Glycogen synthase kinase 3 beta; VEGFA: Vascular endothelial growth factor; ZNF143: Zinc finger protein 143; E6-AP: E6-associated protein; HMGB3: High mobility group box 1 protein; IKBKB: Inhibitor of nuclear factor kappa B kinase subunit beta; CDK2: Cyclin dependent kinase 2; ZEB1: Zinc finger E-box binding homeobox 1; EMT: Epithelial–mesenchymal transition; TGFBI: Transforming growth factor beta induced; MSH2: MutS homolog 2; HMGB1: High mobility group box 1; CCND2: Cyclin D2; SOX9, SRY-box transcription factor 9; PTEN: Phosphatase and tensin homolog; PI3K: Phosphatidylinositol 3-kinase; EGFR: Epidermal growth factor receptor; HDGF: Heparin binding growth factor; LDHA: Lactate dehydrogenase A; CDC25A: Cell division cycle 25A; NHEJ: Non-homologous end joining; HIF-1α: Hypoxia inducible factor 1, alpha subunit; LCN2: Lipocalin 2; CESC: Cervical squamous cell carcinoma and endocervical adenocarcinoma.

**TABLE 3:** Downregulated lncRNAs modulating the therapy resistance of cervical cancer.

Function	Medicine	lncRNA	Target	Mechanism	Ref.
Chemo-resistance	Cisplatin	TRIM52-AS1	-	Inhibited by miR-96–5p, which promotes IGF2BP2 to enhance cancer cell resistance to cisplatin	Wu et al. (2022)
		MEG3	miR-21	Downregulates miR-21 to promote PTEN, enhancing cisplatin sensitivity in cancer cells	Du et al. (2022)
		CASC2	miR-21	Downregulates miR-21 to promote PTEN and inhibit p-AKT, enhancing cisplatin sensitivity in cancer cells	Feng et al. (2017)
		GAS5	miR-21	GAS5 is transcriptionally regulated by p-STAT3 and downregulates miR-21 to promote PDCD4, enhancing cisplatin sensitivity	Fang et al. (2020)
				Downregulates miR-21 to promote PTEN and inhibit Akt phosphorylation, enhancing cisplatin sensitivity	Wen et al. (2017)
				Downregulates miR-21 to promote TIMP3 and PDCD4, inhibiting malignancy and enhancing cisplatin-induced apoptosis	Yao et al. (2019)
Radio-resistance	-		miR-106b	Downregulates miR-106b to promote IER3, enhancing radio-sensitivity	Gao et al. (2019)

Abbreviations: IGF2BP2: Insulin-like growth factor 2 mRNA-binding protein 2; PTEN: Phosphatase and tensin homolog; p-AKT: Phospho-Akt; p-STAT3: Phospho-Signal transducer and activator of transcription 3; PDCD4: Programmed cell death 4; Akt: Protein kinase B; TIMP3: Tissue inhibitor of metalloproteinase 3; IER3: Immediate early response 3.

### 3.1 lncRNAs in chemotherapy

Chemotherapy is a systemic treatment that uses anticancer drugs to kill cancer cells or inhibit their growth, playing a crucial role in the therapy of CC (Gopu et al., 2021). Chemotherapy is primarily used for locally advanced, recurrent, or metastatic CC, and is combined with radiotherapy (Zhou Y. et al., 2022).

For CC treatment, key chemotherapy drugs include platinum-based agents (cisplatin, carboplatin), taxanes (paclitaxel, docetaxel), topoisomerase inhibitors, and other cytotoxic agents (Chen et al., 2024). First-line therapy for patients with persistent, recurrent, or metastatic CC typically involves platinum-based chemotherapy combined with immune checkpoint inhibitors or anti-angiogenic agents (Monk et al., 2023). Second-line therapies use topoisomerase inhibitors, cemiplimab, tisotumab vedotin, or single-agent conventional chemotherapy for recurrent or metastatic CC (Vergote et al., 2024). Mechanistically, chemotherapy drugs for CC commonly kill cancer cells through damaging DNA, disrupting the cell cycle, and inhibiting microtubule function, etc (Tilsed et al., 2022). For instance, platinum drugs form DNA cross-links, induce apoptosis and disrupting transcription (Zhang C. et al., 2022). Taxanes stabilize microtubules, preventing mitotic spindle formation and inducing cell cycle arrest (Yim et al., 2004). For high-risk early-stage, cisplatin-based concurrent chemoradiotherapy is standard (Zhu et al., 2025), while metastatic or recurrent disease uses paclitaxel combined with platinum-based chemotherapy plus bevacizumab (Wang X. et al., 2025). However, chemotherapy for CC carries side effects including hematologic toxicity, gastrointestinal toxicity, neurotoxicity, and genitourinary toxicity, with hematologic toxicity being the most common adverse reaction (Meixner et al., 2024). Furthermore, drug resistance severely limits the therapeutic efficacy of chemotherapy. Resistance mechanisms involve multiple aspects, including regulating glucose metabolism reprogramming, participation in anti-apoptotic and anti-autophagy pathways, epigenetic modulation, and promoting drug efflux, etc (Bukowski et al., 2020; Trujano-Camacho et al., 2025). Multiple studies have confirmed that abnormal lncRNA expression in CC is closely associated with these resistance mechanisms (Zhou M. et al., 2022).

Upregulated lncRNAs, such as linc00958 (Tian et al., 2022a), SOX21-AS1 (Tian et al., 2022b), and BCYRN1 (Sun et al., 2022), promote drug resistance by sponging specific miRNAs (e.g., miR-185-5p, miR-9-3p) to upregulate target genes and activate oncogenic pathways. For example, linc00958 was validated to upregulate remodeling and spacing factor 1 (RSF-1) as ceRNA of miR-185-5. Highly expressed RSF-1 activating the AKT1/GSK3β signaling pathway. The proposed linc00958/miR-185-5p/RSF-1 axis enhances the survival capacity of CC cells and promotes VEGFA secretion and tube formation in cisplatin-resistant SiHa/DDP cells (Tian et al., 2022a). NEAT1 acts as a ceRNA to sponge miR-34a, which targets and inhibits glycolytic enzyme lactate dehydrogenase A (LDHA). The release of LDHA inhibition promotes enhanced cellular glycolysis, thereby conferring 5-Fu resistance in CC cells (Shao et al., 2021). Although direct targeting LDHA by miR-34a has been revealed in other gynecologic tumors, the role of NEAT1 as a ceRNA that competitively liberates LDHA from miR-34a repression in CC remains unexplored. Meanwhile, overexpressed AFAP-AS1 directly inhibit miR-7-5p, thereby inducing the phosphorylation of AKT and MAPK, boosting CC progression and promoting gemcitabine tolerance (Wang C. et al., 2024). However, pharmacologic inhibition is needed to confirm that the AFAP1-AS1/miR-7-5p/EGFR–AKT/MAPK axis drives CC, and the role of AFAP1-AS1/miR-7-5p/EGFR axis in gemcitabine resistance awaits *in-vivo* validation. In addition to downregulating the expression of miR-29b, HOTAIR induces cisplatin, paclitaxel and docetaxel resistance in HeLa and SiHa cancer cells by enhancing EMT, expanding the cancer stem cell pool, and interfering with cisplatin-induced DNA damage repair (Zhang W. et al., 2022). Moreover, UCA1 upregulates the expression of inhibitor of nuclear factor kappa B kinase subunit beta (IKBKB) protein and reduces the pro-apoptotic protein BCL2 interacting protein 3 (BNIP3), ultimately enhances cancer cell proliferation, migration, and invasion capabilities while inhibiting apoptosis, leading to cisplatin resistance (Wang B. et al., 2025). The mechanism by which UCA1 regulates cisplatin resistance needs to be clarified. Mechanistic and cell-line validation studies are warranted to test the hypotheses that UCA1/miR-195–5p/IKBKB drives NF-κB nuclear translocation, downregulates BNIP3, and modulates BNIP3Δex2 splicing in cisplatin-resistant CC.

In contrast, downregulated lncRNAs like MEG3 (Du et al., 2022), CASC2 (Feng et al., 2017), and GAS5 (Fang et al., 2020), improve drug sensitivity by inhibiting miRNAs and activating tumor suppressors (e.g., phosphatase and tensin homolog (PTEN), programmed cell death 4 (PDCD4)), highlighting their potential as therapeutic targets to reverse chemo-resistance. TRIM52-AS1 functions as a crucial ceRNA that sequesters miR-96-5p, thereby preventing its oncogenic targeting of insulin like growth factor 2 mRNA binding protein 2 (IGF2BP2). Depressed TRIM52-AS1 expression consequently elevates IGF2BP2 levels and promotes cisplatin resistance (Wu et al., 2022). However, whether miR-96-5p-regulated lncRNAs or other TRIM52-AS1 targets modulate cisplatin resistance in CC remains untested across cell lines and *in vivo*. MEG3 similarly exerts its chemosensitizing effects through competitive binding of miR-21, which restores tumor suppressor PTEN expression and inactivates Akt signaling (Du et al., 2022). CASC2 operates via an analogous ceRNA mechanism, suppressing miR-21 to upregulate PTEN and attenuate pAkt activation, and its decreased expression correlates with poor prognosis and reduced cisplatin responsiveness in CC patients (Feng et al., 2017). Meanwhile, GAS5 downregulation independently predicts cisplatin resistance and adverse outcomes, with experimental silencing decreasing PCDC4 and TIMP3, both downstream proteins of the STAT3-mediated pathway, thereby promoting malignant phenotypes enhanced proliferation, migration, and invasion (Yao et al., 2019). However, more studies are needed to investigate the mechanism of GAS5 in cisplatin-resistance in CC. In summary, regulating lncRNAs to overcome drug resistance highlights a novel promising target for the treatment of chemo-resistant CC.

### 3.2 lncRNAs in radiotherapy

Radiotherapy is an essential component of CC, applicable across all stages, and particularly effective in achieving radical cure for locally advanced cases (Chino et al., 2020). It affects the cancer in numerous ways. High-energy rays can directly disrupt the DNA structure of cancer cells, preventing them from continuing to divide and proliferate, ultimately leading to cell death (Liu and Ai, 2024). Additionally, radiotherapy promotes the decomposition of water molecules within the body, generating reactive molecules like free radicals, which indirectly inducing apoptosis or necrosis (Chun et al., 2002). Radiation also participate in antigen-related immune responses, overcomes the tumor’s methods of immune evasion via induction of chemokine and cytokine release and increased tumor vascular extravasation, allowing for increased density of tumor-infiltrating lymphocytes in the tumor parenchyma (Feng et al., 2020).

Currently, concurrent chemoradiotherapy employs platinum-based drugs combined with paclitaxel, which attributes to complementary inhibition of homologous recombination repair and G2/M checkpoint abrogation, enhancing ionizing radiation-induced mitotic catastrophe (Yeshe and Li, 2024). Additionally, radiosensitizers such as nimorazole target hypoxic tumor cells (Siemann and Horsman, 2015). Hypoxia-driven radioresistance is countered by the 2-nitroimidazole nimorazole, which mimics oxygen and fixes radiation-induced DNA free-radical damage, thereby reducing cancer cell repair (Olender et al., 2018).

Despite advances in radiotherapy for CC over years, challenges such as insufficient sparing of organs at risk, notable acute and late toxicities, and limited long-term outcome evidence remain (Liu et al., 2025). While these techniques improve dose conformity, balancing target coverage with normal tissue protection and ensuring accessibility remains clinically challenging. Furthermore, emerging studies highlighted that lncRNAs primarily modulate CC cells’ sensitivity to radiotherapy by influencing DNA damage repair, cell cycle regulation, apoptosis, glycolysis, and interactions with miRNAs (Xie et al., 2022).

Upregulated lncRNAs contribute to radio-resistance by downregulating miRNAs, promoting DNA repair, enhancing cell proliferation, and reducing apoptosis. For example, linc00662 promotes the expression of the cell cycle regulator CDC25A by binding to miR-497-5p. This promotes proliferation and migration in CC cells, ultimately reducing their sensitivity to radiotherapy (Wei et al., 2020). LINP1 directly binds to core proteins of the non-homologous end-joining (NHEJ) pathway, forming functional complexes that enhance NHEJ-mediated double-strand break repair activity. This promotes DNA damage repair, inhibits apoptosis, and consequently increases radiation resistance in CC (Wang et al., 2018a). Meanwhile, HOTAIR restores cellular vitality by upregulating HIF-1α expression while reducing apoptosis, thereby regulating tumor cell drug resistance (Li et al., 2018). However, the mechanism by which HOTAIR interacts with HIF-1α in CC cells undergoing radiotherapy warrants further investigation.

Conversely, downregulated lncRNAs such as GAS5 sensitize cancer cells to radiation by regulating the miR-106b/IER3 axis, indicating that targeting these lncRNAs could improve radiotherapy efficacy in CC (Gao et al., 2019). These converging lncRNA-driven mechanisms thus constitute critical molecular vulnerabilities that may be exploited to overcome radioresistance in CC. Furthermore, The link between anti-tumor drugs and lncRNAs still needs to be fully studied and validated. Understanding such mechanisms is critical for effective application of these drugs.

### 3.3 lncRNAs in immunotherapy

High-risk HPV papillomavirus (hrHPV) types, particularly HPV-16 and HPV-18, are highly associated with CC (Wei et al., 2024). hrHPV oncoproteins E6/E7 orchestrate a permissive immune microenvironment by up-regulating PD-L1, skewing tumor-associated macrophages toward the M2 phenotype, and inducing IDO1-mediated tryptophan depletion (Li et al., 2025). In addition, HPV16 promote the overexpression of ERAP1, impairing CD8^+^ T-cell recognition, promoting immune escape in CC (Steinbach et al., 2017). HPV infection can also downregulate MHC class I molecule expression, diminishing the ability of tumour cells to be recognised by T cells (Evans et al., 2022). Immunotherapy modulates HPV infection-associated immune evasion through multiple mechanisms, restoring the body’s capacity for immune surveillance and clearance of HPV-positive tumour cells. For example, therapeutic HPV vaccines can activate antigen-presenting cells, inducing the production of HPV-specific CD8^+^ T cells. These T cells recognise and eliminate tumour cells expressing E6/E7, thereby reshaping the tumour immune microenvironment and enhancing the sustained efficacy of immunotherapy (Fert et al., 2024). Meanwhile, immunotherapy strategies can upregulate MHC expression, enhance antigen presentation, and improve the immune system’s capacity to recognise HPV-infected cells (Shamseddine et al., 2021).

Therefore, immunotherapy has emerged as a promising treatment approach for HPV-related malignancies. Immune checkpoint inhibitors (ICIs) have shown efficacy in enhancing the immune response against HPV-associated tumors by blocking proteins that allow cancer cells to evade immune surveillance (Ghosh et al., 2025). Currently approved by U.S. Food and Drug Administration (FDA) drugs for HPV-related cancers include pembrolizumab (Keytruda), which was applied for stage III-IVA CC in combination with chemoradiotherapy (Ogasawara and Hasegawa, 2025). Other drugs such as atezolizumab and durvalumab play a crucial role in CC treatment by restoring immune recognition of tumor cells (Ogasawara and Hasegawa, 2025). Meanwhile, clinical trials demonstrate that adding ICIs to standard therapy significantly improves progression-free survival and overall survival compared to standard therapy alone (Tarhini et al., 2022). For example, pembrolizumab combined with chemotherapy and bevacizumab has shown significant improvements in both PFS and OS in the KEYNOTE-826 trial (Kim et al., 2025).

In immunotherapy strategies, the appearance of ICIs raised new hope for CC treatment. Research has identified multiple lncRNAs that are closely associated with immune checkpoint molecule expression. lncRNAs modulate immune evasion by upregulating PD-L1 or regulating ceRNA network (Zhou W. Y. et al., 2019). For instance, MALAT1 and linc00473 both increase PD-L1 expression by binding to miRNAs, thereby affecting CC cell’s sensitivity to immunotherapy (Trujano-Camacho et al., 2025). Moreover, differentially expressed genes in m^6^A-associated lncRNA clustering patterns are significantly associated with PD-L1 expression and the PD-1 checkpoint pathway. This pathway inhibits T cell activation, leading to immune cell functional exhaustion and subsequently triggering immune resistance (Wang et al., 2023). Meanwhile, mitochondrial oxidative stress-associated lncRNAs regulate TME and predict immunotherapy response, which is of core significance for providing reliable predictive biomarkers and potential therapeutic targets for CC immunotherapy (Wu et al., 2025). In CC, the specific therapy for PD-L1-positive is relatively new, so the resistance mechanism to this therapy is still to be studied. Meanwhile, research on the synergistic effects of lncRNA in immune therapy is gaining significant progress. These advancements provide crucial theoretical foundations and technical support for developing novel lncRNA-based therapy strategies.

### 3.4 lncRNAs in targeted therapy

Targeted therapy specifically targets molecular aberrations in cancer cells that help them grow, divide, and spread, while minimizing damage to healthy tissues. The development of targeted therapeutic agents for CC has primarily focused on anti-angiogenic therapy and immune checkpoint inhibitors (Wang et al., 2020). For CC, bevacizumab (avastin, genentech), pembrolizumab (keytruda, merck), and lenvatinib (lenvima, eisai) have been approved by the FDA for selected patients with recurrent, metastatic, or high-risk diseases (Wang et al., 2020).

Antiangiogenic therapies are developed by inhibiting target signaling pathways at different points. For example, bevacizumab, an anti-VEGF monoclonal antibodies, has been currently approved by FDA as combination treatment and/or maintenance treatment for selected patients with persistent, recurrent, or metastatic CC (Monk et al., 2009). Bevacizumab exerts its anti-tumor effects by binding to and neutralizing vascular endothelial growth factor A (VEGF-A) related signaling pathways (e.g., PI3k/AKT, ERK/MAPK), thereby inhibiting tumor angiogenesis, leading to delayed tumor progression (Loizzi et al., 2017). Immune checkpoint inhibitors such as pembrolizumab is approved by FDA for treating advanced or recurrent CC (Frenel et al., 2017). Pembrolizumab targets and blocks the interaction between the PD-1 receptor on the surface of T cells and its ligands PD-L1/PD-L2, thereby restoring the antitumor immune response (Colombo et al., 2021). Lenvatinib is a multikinase inhibitor targeting VEGFR, FGFR, PDGFR, RET, and KIT (Hao and Wang, 2020). Lenvatinib blocks their downstream signaling pathways (MAPK, PI3K/AKT, etc.), which suppresses tumor cell proliferation, migration, and angiogenesis (Okamoto et al., 2015).

lncRNAs have emerged as critical regulators of target therapy resistance in CC, affecting the efficacy of drugs through regulating related signaling pathways (Manzo-Merino et al., 2014). For example, HOTAIR enhances chemo-resistance by inhibiting miR-29b, thereby activating PTEN/PI3K pathway, promoting cell proliferation and migration (Zhang W. et al., 2022). Overexpression of HOTAIR enhances the transcriptional activity of Wnt/β-catenin, preventing ICRT14 from effectively blocking the Wnt pathway and leading to drug resistance (Trujano-Camacho et al., 2021). Meanwhile, highly expressed PVT1 epigenetically promotes RAF1, leading to sustained activation of the MEK-ERK signaling cascade and inducing EMT (Trujano-Camacho et al., 2021). The complex regulatory networks involving lncRNAs and key signaling molecules represent potential therapeutic targets for overcoming resistance and improving treatment outcomes in CC.

### 3.5 lncRNAs in combined therapy

Recent multimodal strategies integrating platinum-based chemotherapy, radiotherapy and PD-1/PD-L1 blockade have extended median overall survival in recurrent CC, and the addition of bevacizumab to chemo-immunotherapy further improves progression-free survival (K et al., 2025). lncRNA MEG3 down-regulates PI3K/AKT/Bcl-2 signaling to sensitize tumour cells to cisplatin, while LINC00426 acts as a competing endogenous RNA for miR-200a-3p, thereby sustaining EMT and radio-resistance (Yang et al., 2022). In addition, lncRNA MDC1-AS enhances combined therapy by binding MDC1 mRNA to prevent RNase degradation, modulates apoptosis-related genes BAX/Bcl-2, and synergizes with oxaliplatin, thereby promoting apoptosis and inhibiting proliferation to boost therapy efficacy (Ye et al., 2021).

Conversely, by integrating lncRNA regulation with immunotherapy, targeted therapy, and chemoradiotherapy, it is anticipated that traditional treatment limitations can be overcome, achieving therapeutic breakthroughs such as multi-targeted synergistic effects and chemoradiotherapy sensitization.

## 4 Clinical management of lncRNAs in CC

Mounting evidence has demonstrated that numerous lncRNAs mediate the therapeutic process in CC, including diagnostic and prognostic biomarkers, and therapeutic target (He et al., 2020).

### 4.1 lncRNAs serve as diagnostic biomarkers

Clinical studies indicate that only approximately 46% of CC patients are diagnosed early, with poor survival in advanced or recurrent cases (Fedewa et al., 2012). Current biomarkers lack specificity, and incidence rises in younger populations (He et al., 2020). lncRNAs, with non-invasive detection, high specificity, stable serum and exosome presence, and tumor progression relevance, are imperative as superior diagnostic biomarkers for CC (Chen et al., 2025b) (Table 4).

**TABLE 4:** lncRNAs as diagnostic biomarkers in cervical cancer.

lncRNA	Regulation	Function	Sensitivity	Specificity	Ref.
MEG3	Downregulated	Downregulated in serum of cervical cancer patients. A potential diagnostic and prognostic biomarker with high accuracy (AUC 0.844 for diagnosis, AUC 0.858 for mortality prediction)	82.76%	75.00%	Zhang J. et al. (2017)
GIHCG	Upregulated	Upregulated in cervical cancer tissues, cell lines and serum	88.75%	87.50%	Liu et al. (2024)
GHET1	Upregulated	A diagnostic biomarker distinguishing cervical cancer from non-tumorous tissues (AUC = 0.874, 95% CI:0.802–0.940)	-	-	Zhang et al. (2019b)
PVT1	Upregulated	A diagnostic biomarker for cervical squamous cell carcinoma (AUC 0.9030, 95% CI: 0.8567–0.9472)	-	-	Wang et al. (2018b)
C5orf66-AS1	Upregulated	Upregulated in cervical cancer relative to matched para-cancer normal samples. Expression increases with tumor stage progression	-	-	Luo et al. (2020)
AC126474	Downregulated	Downregulated in cervical cancer, involved in keratinization and immune-related pathways	-	-	
CCAT2	Upregulated	Elevated in serum of cervical cancer patients, correlates with FIGO stage, SCC-Ag and lymph node metastasis. Serves as a panel with CA125 and SCC for high diagnostic accuracy (AUC 0.897, 95% CI: 0.862–0.933)	73.33%	87.00%	Cao et al. (2022)
DLEU1	Upregulated	Elevated in serum exosomes of cervical cancer patients. Combined with CA-125 and SCC, it achieves an AUC of 0.878 (95% CI: 0.837–0.920) for cervical cancer diagnosis	65.7%	94.0%	Chen et al. (2025b)
DLX6-AS1	Upregulated	High diagnostic accuracy for CC vs. controls (AUC 0.892) and CC vs. CIN (AUC 0.831)	78.1% (CC vs. controls); 75.4% (CC vs. CIN)	88.2% (CC vs. controls); 71.8% (CC vs. CIN)	Ding et al. (2021)
HOXC13-AS	Upregulated	Highly expressed in cervical cancer tissues and cell lines	-	-	Gu et al. (2025)
FALEC	Upregulated	Distinguishes cervical cancer patients from normal individuals (AUC = 0.91) and differentiates Ib-IIa from IIb-IIIa stages (AUC = 0.88 and 0.97 respectively)	-	-	Naizhaer et al. (2019)
HOXA-AS3	Upregulated	Elevated in cervical cancer tissues and cells compared with normal controls, verified by TCGA and GTEx databases	-	-	Xu et al. (2022)
DLEU1	Upregulated	Distinguishes CC from controls (AUC = 0.951), differentiates HPV+ from HPV- CC patients (AUC = 0.867), and screens HPV+ from HPV- controls (AUC = 0.757)	87.8% (for distinguishing HPV+ from HPV- CC patients)	81.6% (for distinguishing HPV+ from HPV- CC patients); 71.4% (for screening HPV+ from HPV- controls)	Dong et al. (2022)
LINC00989, LOC105374768	Downregulated	Part of a panel with other RNAs to distinguish normal and cancer groups	90%	75%	Cho et al. (2021)
PCAT1	Upregulated	Moderate diagnostic value with AUC 0.79 (95% CI: 0.73–0.85)	79%	69%	Bazmi et al. (2025)
PCAT2			74%	62%	
PCAT3			69%	66%	
Combined PCAT1, PCAT2, PCAT3			80%	71%	

Abbreviations: AUC: area under curve; CI: confidence interval; SCC-Ag: Squamous cell carcinoma associated antigen; CA-125: Carbohydrate antigen 125; SCC: squamous cell carcinoma antigen; CIN: cervical intraepithelial neoplasia.

HPV diagnostic enables earlier, more sensitive detection of CC and its precursors than cytology alone, and allows evidence-based risk stratification that reduces unnecessary follow up test and overtreatment (Magdi et al., 2021). Research confirms that plasma MEG3 methylation levels in hr-HPV-positive patients are significantly higher than in HPV-negative patients. Plasma MEG3 methylation demonstrates excellent diagnostic efficacy for HPV infection and offers high practicality (Zhang J. et al., 2017). Meanwhile, OIS1 expression is significantly lower in tumor tissues of HPV-positive CC patients, whereas this difference is barely discernible in HPV-negative patients. Therefore, it may serve as a diagnostic biomarker for HPV-positive CC (Zhou D. et al., 2019). Due to their excellent stability across various sample types (such as FFPE, plasma, and other bodily fluids) and their accessibility through non-invasive methods, lncRNAs represent highly promising diagnostic biomarkers (Casarotto et al., 2020).

For cancer diagnostic, GIHCG is significantly upregulated in CC tissues, cell lines, and serum, showing high diagnostic accuracy via receiver operating characteristic analysis and oncogenic effects by repressing miR-200b (Zhang et al., 2019c). In serum, exosomal DLEU1 is elevated in CC patients, associated with tumor size, International Federation of Gynecology and Obstetrics (FIGO) stage, and lymph node metastasis, and combined detection with tumor biomarkers CA-125 and SCC enhances diagnostic efficiency (Chen et al., 2025b). Similarly, PVT1 highly expressed in serum. Study implied that it distinguishes patients from healthy individuals with diagnostic efficacy. Moreover, its stability in plasma ensures clinical applicability as a non-invasive indicator (Sun et al., 2018). HOTAIR exhibits higher diagnostic performance in vaginal discharge than serum, correlating with tumor malignancy and decreasing post-surgery (L et al., 2023). SCC-Ag predicts lymph node metastasis in CC but with low sensitivity and specificity, limiting its diagnostic value (Wu et al., 2019). In CC tissues, PCAT1, PCAT2, and PCAT3 are more abundant, with PCAT1 showing potential as an effective diagnostic marker through regulating oncogenic pathways (Bazmi et al., 2025). In addition, BCRT1-based biosensors enable highly sensitive and specific CC detection via exosomal BCRT1, offering a novel diagnostic method (Cong et al., 2025). SNHG17 and miR-375–3p effectively distinguish CC patients from healthy controls (Cao et al., 2021). However, there are challenges including standardized detection methods and thresholds, the need for multi-biomarker combinations to improve sensitivity, deepened molecular mechanism research, and large-scale clinical validation for translational application (He et al., 2020).

In summary, lncRNAs exhibit significant potential as diagnostic biomarkers for CC. With standardized protocols, multi-biomarker panels, and deepened mechanism studies, lncRNAs holds promise for improving early and precise diagnosis of colorectal cancer, thereby enhancing survival rates for patients worldwide.

### 4.2 lncRNAs serve as prognostic biomarkers

Beyond diagnostic applications, lncRNAs exhibit significant value as prognostic indicators in CC, with their expression levels correlating closely with tumor aggressiveness, metastatic potential, and patient survival (Table 5). These associations stem from their functional roles in driving therapeutic resistance and malignant progression.

**TABLE 5:** lncRNAs as prognostic biomarkers in cervical cancer.

lncRNA	Regulation	Function	Ref.
MEG3	Downregulated	Downregulated in serum of cervical cancer patients. A potential diagnostic and prognostic biomarker with high accuracy	Zhang J. et al. (2017) Wan and Zhao (2020)
RUSC1-AS1	Upregulated	Part of 8-lncRNA signature predicting poor prognosis with high AUC (>0.75 in TCGA, >0.6 in GEO)	Zhong et al. (2021)
LINC01990			
LINC01411			
LINC02099			
H19			
LINC00452			
ADPGK-AS1			
C1QTNF1-AS1			
SOX21-AS1	Upregulated	High expression of SOX21-AS1 correlates with poor prognosis of cervical cancer patients	Zhang et al. (2019d), Wang et al. (2019b)
GHET1	Upregulated	An independent unfavorable prognostic biomarker associated with advanced clinical stage, lymph node/distant metastasis, poor histological grade, and shorter overall survival	Zhang et al. (2019b)
PVT1	Upregulated	A prognostic biomarker with high serum levels indicating shorter overall survival	Wang et al. (2018b)
LINC00511	Upregulated	High expression indicates poor overall survival and serves as an independent poor prognostic factor	Yu et al. (2019)
AFAP1-AS1	Upregulated	High expression and hypomethylation associated with shorter overall survival in cervical cancer patients	Bo et al. (2019)
C5orf66-AS1	Upregulated	Associated with poor prognosis	Luo et al. (2020)
AC126474	Downregulated	Associated with favorable prognosis.Downregulated in cervical cancer, involved in keratinization and immune-related pathways	
CCAT2	Upregulated	Correlates with clinicopathological features related to poor prognosis (FIGO stage, SCC-Ag, lymph node metastasis)	Cao et al. (2022)
DLEU1	Upregulated	High expression in cervical cancer patients is associated with poor prognosis, including reduced overall survival and disease-free survival	Chen et al. (2025b)
DLX6-AS1	Upregulated	High expression correlates with shortened OS/RFS and higher relapse risk. Serves as an independent prognostic indicator for CC.	Ding et al. (2021)
AATBC	Upregulated	An independent prognostic indicator. High expression predicts adverse overall survival in cervical cancer patients	Liu et al. (2023)
HOXC13-AS	Upregulated	Highly expressed in cervical cancer tissues and cell lines	Gu et al. (2025)
FALEC	Upregulated	Correlated with tumor size, FIGO stage, lymph node metastasis. High expression predicts shorter overall survival	Naizhaer et al. (2019)
HOXA-AS3	Upregulated	An independent prognostic factor (HR = 3.224, 95% CI:1.399–7.431, p = 0.006) with low expression associated with better patient survival	Xu et al. (2022)
DLEU1	Upregulated	Independently predicts overall survival of total CC patients (HR = 2.437, 95% CI = 1.612–3.329, p = 0.004) and HPV + CC patients, with high expression indicating poor prognosis	Dong et al. (2022)
TDRG1	Upregulated	High expression correlates with shorter overall survival	El-Lateef et al. (2024)
AL441992.1 LINC01305 AL354833.2 CNNM3-DT SCAT2 AL354733.3 AC009902.2	Downregulated	Contributes to the signature with high prognostic accuracy (AUC 0.724–0.824 for 1–5 years)	Liu et al. (2022)
AC008124.1 RPP38-DT AC073529.1	Downregulated	An independent prognostic signature predicting overall survival (AUC: 0.708 in training cohort, 0.668 in testing cohort)	Zhang et al. (2022c)
AC015922.2	Upregulated		
SCAT1 AC119427.1 MIR100HG MIAT AP003774.2 AC004540.2	Upregulated	A 9-lncRNA signature predicts overall survival (high-risk patients have shorter OS)	Lang et al. (2022)
AC010996.1 AL583856.2	Downregulated		
AC009097.2	-		

Abbreviations: SOX21-AS1: SRY-box transcription factor 21-antisense RNA, 1; SCC-Ag: Squamous cell carcinoma associated antigen; OS/RFS: Overall survival/Recurrence-free survival; HR: Hazard ratio; CI: Confidence interval; AUC: Area under curve.

Multiple lncRNAs show robust prognostic utility by tracking key clinically pathological features. Linc00511 overexpression associates with advanced FIGO stage, larger tumor size, and lymph node metastasis, serving as an independent predictor of poor outcomes (Yu et al., 2019). Similarly, FAM83H-AS1 upregulation during early carcinogenesis correlates with reduced overall survival through modulation of HPV16 E^6^ oncogenic activity (Barr et al., 2019). In circulating biofluids, plasma MEG3 methylation (diagnostic AUC 0.844) and serum exosomal DLX6-AS1 (diagnostic AUC 0.892) not only distinguish patients from healthy controls but also predict metastatic progression and shortened relapse-free survival, underscoring their dual diagnostic-prognostic value (Huang et al., 2021).

Critically, the prognostic power of these lncRNAs often stems from their direct roles in therapy resistance. PVT1 overexpression predicts chemoresistance and radiotherapy failure by activating NF-κB signaling and promoting EMT (Fox et al., 2020). Conversely, downregulation of tumor-suppressive lncRNAs like GAS5 and CASC2 independently predicts cisplatin resistance and inferior survival, as their loss releases miR-21-mediated inhibition of PTEN and activates pro-survival pathways (Fang et al., 2020). These findings demonstrate that lncRNA-mediated resistance is not merely a molecular event but a clinically actionable predictor of treatment failure, paving the way for risk-adapted therapeutic strategies.

Despite this promise, several hurdles remain. Prognostic lncRNA signatures require validation across diverse patient cohorts and standardized cut-off values to ensure reproducibility. Current studies predominantly focus on single lncRNAs rather than multi-marker panels, which may better capture the heterogeneity of CC progression. Moreover, the functional basis underpinning many prognostic associations remains incompletely elucidated, limiting their integration into risk-stratification algorithms. Nevertheless, as circulating lncRNAs can be longitudinally monitored, they hold unique potential for dynamic risk assessment and early detection of resistance emergence. Future efforts should prioritize prospective clinical studies to establish robust, validated lncRNA-based prognostic models that can guide treatment intensification or de-escalation, ultimately moving toward personalized CC management.

### 4.3 lncRNAs serve as therapeutic targets

**FIGURE 4:**
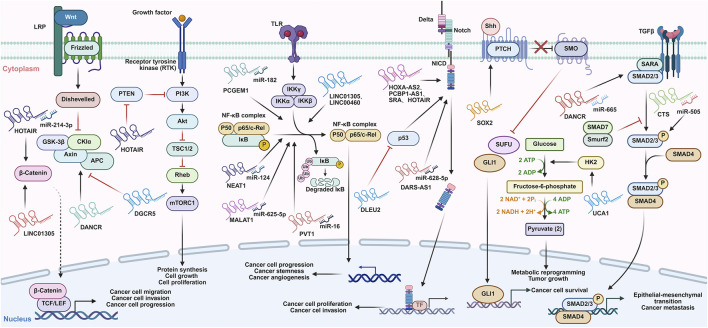
lncRNAs are involved in various signaling pathways involved in the cervical cancer progression. lncRNAs can regulate the activation of the Wnt, PI3K/Akt, NF-κB, Notch, HK2/glycolytic, Hedgehog, and SMAD signaling pathways via different mechanisms. These signaling pathways play roles in the promotion or inhibition of cervical cancer progression. Notably, these signaling pathways can also regulate the expression of some IncRNAs, forming regulation loops to affect the occurrence and development of cervical cancer. Abbreviations: LRP: LDL receptor related protein; GSK-3β: Glycogen synthase kinase 3 beta; CKIα: Casein kinase Iα; APC: Adenomatous polyposis coli; TCF/LEF: T-cell factor/lymphoid enhancer-binding factor; PI3K: Phosphatidylinositol 3-kinase; Akt: Protein kinase B; TSC1/2: Tuberous sclerosis complex 1/2; Rheb: Ras homolog enriched in brain; mTORC1: mTOR complex 1; IKKγ: Inhibitor of kappa B kinase γ; IκB: Inhibitor of NF-κB; TLR: Toll like receptor; NICD: Notch intracellular domain; Shh: Sonic hedgehog; PTCH: Patched 1; SMO: Smoothened; SUFU: SUFU negative regulator of hedgehog signaling; GLI1: GLI family zinc finger 1; SARA: Smad anchor for receptor activation; Smurf2: SMAD specific E3 ubiquitin protein ligase 2; HK2: Hexokinase 2.

The therapeutic potential of lncRNAs is under investigation. Studies have shown that numerous crucial signaling pathways such as Wnt/β-Catenin, PI3K/AKT, NF-κB and Notch are found to be frequently abnormally regulated by lncRNAs (Figure 4), which offers significant clinical implications for the clinical management of CC.

#### 4.3.1 lncRNAs in Wnt signaling pathway

The Wnt protein family interacts as ligand and receptor complexes to stabilize free β-catenin in the cytoplasm (MacDonald et al., 2009). Subsequently, accumulated β-catenin translocates to the nucleus, promoting the expression of target genes to influence tumor progression (Katoh and Katoh, 2017). Previous research has summarized how lncRNAs are involved in the Wnt/β-catenin signaling pathway to affect the progression of CC. For instance, DANCR acts as an oncogenic lncRNA in CC by activating the Wnt/β-catenin signaling pathway (Tian et al., 2020). linc01305, mainly distributed in exosomes, is upregulated in CC cells and tissues with high CD44 levels. It activates the Wnt pathway by upregulating β-catenin, TCF7, and CCND2, thereby promoting CC progression and maintaining cancer stemness (Huang et al., 2021; Wang et al., 2015). Meanwhile, HOTAIR forms a regulatory axis with miR-214-3p in HPV16-positive CC. It up-regulates β-catenin expression by sponging miR-214-3p, activating the canonical Wnt/β-catenin pathway to induce malignant phenotypes of CC cells (Zhou et al., 2021). On the contrary, DGCR5 functions as a tumor suppressor in CC. Its silencing activates the Wnt signaling pathway to enhance cell colony formation and inhibit apoptosis, while its overexpression suppresses CC cell migration, invasion, and progression (Liu et al., 2019).

Wnt signaling is a key cascade closely associated with cancer progression and is highly activated in CC cells. Therefore, characterization of the complex relationship between lncRNAs and target genes were needed to further carried out, help to improve the early diagnosis and treatment of CC.

#### 4.3.2 lncRNAs in PI3K/Akt signaling pathway

The PI3K/Akt signaling pathway consists of the regulatory subunit p85 and the catalytic subunit p110 (Fox et al., 2020). Upon receiving signals from growth factor receptors, the pathway activates serine/threonine kinase AKT, which then regulates cell proliferation, differentiation, and apoptosis via phosphorylating downstream substrates (Hoxhaj and Manning, 2020). lncRNAs are closely involved in regulating the PI3K/Akt pathway in CC. For example, lncRNA ANRIL is significantly upregulated in CC, correlating with advanced FIGO stage, lymph node metastasis, and poor overall survival. The knockdown of ANRIL inhibits CC cell proliferation and metastasis *in vitro* by inhibiting the PI3K/Akt pathway (Zhang et al., 2018; Zhang D. et al., 2017). HOTAIR mediates positive crosstalk between the Wnt/β-catenin and PI3K/AKT pathways in CC via a feedback loop where HIF1α induces HOTAIR overexpression, sustaining Wnt/β-catenin pathway activity and activating PI3K/AKT by methylating the PTEN promoter (Trujano-Camacho et al., 2024). Additionally, HOTAIR promotes CC chemoresistance by facilitating EMT through the miR-29b/PTEN/PI3K axis, while transfection of miR-29 b mimics significantly downregulates PI3K (Zhang W. et al., 2022).

Therefore, future research should delve into the cell type-specific mechanisms regulating the PI3K/Akt pathway and further validate the prevalence of the lncRNA-PI3K/Akt regulatory axis in clinical samples, thereby providing more robust evidence for its clinical application.

#### 4.3.3 lncRNAs in NF-κB signaling pathway

The NF-κB signaling pathway is frequently dysregulated in CC and modulated by lncRNAs to drive tumor progression (Zhao et al., 2021). LINC01305 is mainly distributed in exosomes, transferred to recipient cells, and binds to RNA-binding protein KHSRP to activate NF-κB and STAT pathways, significantly promoting CC progression and enhancing cancer stemness (Huang et al., 2021). Linc00460 promotes CC angiogenesis by activating the NF-κB-VEGFA axis, as its knockdown attenuates human umbilical vein endothelial cell angiogenesis, with vascular endothelial growth factor A (VEGFA) transcription stimulation being the key mechanism (Li and Zhu, 2023). lncRNA NEAT1 is upregulated in CC, promotes cell proliferation, migration, invasion, EMT, and activates the NF-κB pathway by inhibiting miR-124 (Shen et al., 2020). MALAT1 acts as a ceRNA for miR-625-5p, which is downregulated in CC and inhibits cell growth by inhibiting NF-κB, thereby contributig to the malignant proliferation of CC cells (Li et al., 2020). PVT1 overexpression accelerates CC cell growth by activating NF-κB, while its effect is regulated by miR-16, which directly interacts with NF-κB (Wang C. et al., 2019). PCGEM1 is significantly upregulated in CC, and its high expression correlates with advanced FIGO stage, metastasis, and poor prognosis. Functionally, PCGEM1 promotes cell proliferation, cycle progression, migration, and invasion while suppressing apoptosis. It acts as a ceRNA for FBXW11 by sponging miR-182, thereby activating the NF-κB pathway (Zhang et al., 2019e).

In conclusion, multiple lncRNAs converge on NF-κB to modulate CC aggressiveness, offering reliable, pathway-focused biomarkers. Future studies should clarify the feedback circuits between lncRNA and NF-κB, and develop lncRNA-targeted therapy tools to safely restore NF-κB homeostasis in CC.

#### 4.3.4 lncRNAs in Notch signaling pathway

Notch signaling transduction, a conserved cell-signaling mechanism, is closely linked to CC progression, including cell proliferation, invasion, migration, and EMT (Zhou B. et al., 2022). lncRNAs exert regulatory effects on the Notch pathway to modulate CC development. lncRNA DLEU2 inhibits Notch pathway activity via p53, promoting CC cell proliferation by accelerating the cell cycle (He M. et al., 2021). HOTAIR modulates Notch-Wnt signaling-related genes, enhancing CC cell proliferation, invasion, and correlating with poor patient survival (Trujano-Camacho et al., 2024; Lee et al., 2016). HOXA-AS2 binds NICD to strengthen its interaction with downstream transcription factor RBP-JK, activating the Notch pathway to promote CC cell proliferation and migration (Wu et al., 2021). PCBP1-AS1 is associated with Notch signaling activation, and its depletion inhibits CC cell proliferation, migration, and invasion (Li et al., 2021). lncRNA SRA upregulates NOTCH1, transcription factor HES1, and oncogene co-activator p300, activating the Notch pathway to induce EMT and promote CC cell proliferation, migration, and invasion (Eoh et al., 2017). DARS-AS1 competitively binds miR-628–5p to upregulate key ligands of the Notch signaling pathway JAG1, activating the Notch pathway to accelerate CC tumorigenesis (Chen et al., 2020).

Collectively, these lncRNAs regulate the Notch pathway through diverse mechanisms, serving as potential therapeutic targets and prognostic markers for CC. Future research should further investigate the mechanisms underlying lncRNA-Notch interactions.

#### 4.3.5 lncRNAs in other signaling pathways

lncRNAs also participate in modulating other pathways involved in chemo-resistance, such as glycolytic, Hedgehog (Hh), and SMAD signaling pathways. The HK2/glycolytic pathway is crucial for regulating cancer radio-resistance, with hexokinase 2 (HK2) as a key mediator (Zhou B. et al., 2022). lncRNA UCA1 promotes radioresistance-associated glycolysis in radioresistant CC cells by regulating HK2, and glycolysis inhibition reverses its effects (Fan et al., 2018). The Hh signaling pathway, vital for tumor progression, is activated in radioresistant CC cells. SOX2, an independent prognostic factor, binds the Hh signaling pathway regulatory factor HHAT to activate Hh signaling, enhancing radioresistance by promoting cell survival and inhibiting apoptosis (Huang et al., 2018). The SMAD signaling pathway, mediated by TGF-β, regulates malignant phenotypes in CC. lncRNA DANCR acts as a ceRNA for miR-665, upregulating TGFBR1 to activate the ERK/SMAD pathway and promote metastasis (Cao et al., 2019). lncRNA-CTS targets miR-505 to activate the TGF/SMAD pathway via upregulating p-SMAD2/3 and tumor-associated macrophage regulatory factors ZEB2, facilitating EMT and metastasis in CC (Feng S. et al., 2019).

lncRNAs demonstrate significant potential in CC therapy, with their dysregulated signaling pathways providing crucial clues for clinical diagnosis. Research into their target mechanisms is vital to bridge gaps in clinical validation. Future studies on these pathways will accelerate the progression of lncRNA therapies from preclinical exploration to translational applications, thereby enhancing treatment outcomes for CC.

## 5 Novel therapeutic approaches for targeting lncRNAs

**FIGURE 5:**
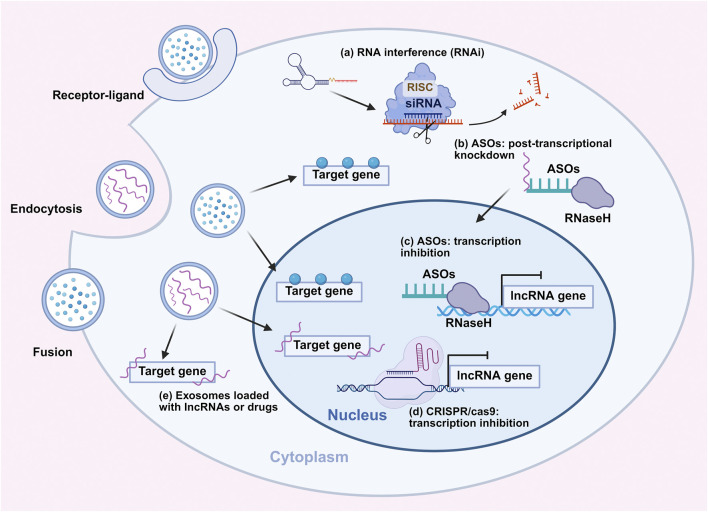
Novel therapeutic strategies targeting lncRNAs in cervical cancer. **(a)** Degradation of the RNA by RNAi. **(b)** ASOs mediate post-transcriptional knockdown of lncRNAs in the cytoplasm via RNase H-dependent degradation. **(c)** ASOs inhibit lncRNA transcription in the nucleus through RNase H-mediated cleavage of nascent lncRNA transcripts. **(d)** CRISPR/Cas9 system exerts transcriptional inhibition of lncRNA genes in the nucleus. **(e)** Exosomes loaded with lncRNAs or drugs deliver therapeutic cargos into cells via endocytosis and fusion, modulating target genes involved in cervical cancer progression. Abbreviations: RNAi: RNA interference; ASO: Antisense oligonucleotides.

Targeting lncRNAs holds great promise as a therapeutic strategy for CC, with antisense oligonucleotides (ASOs), CRISPR/Cas9 system, and exosome-mediated delivery leading the research progress (Figure 5) (Parashar et al., 2022). These methods leverage distinct mechanisms to regulate lncRNA expression, addressing the limitations of conventional therapies by targeting lncRNAs’ roles in CC carcinogenesis (Yang and Al-Hendy, 2022).

### 5.1 RNAi

RNAi offers potent therapeutic strategies for targeting oncogenic lncRNAs in CC. Small interfering RNAs (siRNAs) and short hairpin RNAs (shRNAs) induce sequence-specific degradation of target lncRNAs through the RISC complex (Parashar et al., 2022). Chemical and delivery optimizations of this therapy strategy improve stability, cellular uptake, and tumor accumulation. In CC, RNAi targeting oncogenic lncRNAs such as NEAT1 suppresses proliferation, migration, and chemoresistance while inducing apoptosis (Geng et al., 2022), which validate RNAi as a novel therapeutic strategy for CC. Further development requires enhanced delivery systems, reduced off-target effects, and combination regimens to advance clinical translation.

### 5.2 ASOs

ASOs are short single-stranded deoxyribonucleotide analogs that bind specific RNA sequences, triggering RNase H1-mediated degradation. Their chemical modifications enhance stability, binding affinity, and safety (Hörberg et al., 2024). In CC, ASOs targeting ASncmtRNA induce massive apoptotic cell death and reduce tumorigenicity (Piergentili and Sechi, 2024), while MALAT1-specific ASO FLM-7523 inhibits MALAT1 expression, blocking its oncogenic effects, thereby enhancing CC cell sensitivity to chemotherapeutic agents and inducing apoptosis (Amodio et al., 2018). These findings position ASOs as promising tools, though clinical translation will require optimizing systemic delivery, evaluating long-term safety profiles, and addressing potential challenges in achieving therapeutic concentrations within tumor tissues. Combination strategies with chemoradiation may be worth exploring to overcome compensatory resistance mechanisms.

### 5.3 CRISPR/Cas9

CRISPR/Cas9, a precise genome-editing tool, targets both coding and non-coding genomic regions (Cong et al., 2013). In CC research, the CRISPRi system represses transcription without double-strand breaks, aiding in identifying lncRNAs such as LH02236 critical for cancer invasion (Kim M. et al., 2024). However, off-target effects, delivery barriers, and immunogenicity still hinder its clinical translation, despite advances in delivery systems like lipid nanoparticles and viral vectors (Wilbie et al., 2019). Refining guide RNA specificity and developing tissue-specific delivery vehicles remain critical next steps. Future iterations may need to combine CRISPRi with epigenetic modulators to overcome the repressive chromatin environments that characterize many resistance-related lncRNAs.

### 5.4 Exosomes

Exosomes serve as natural carriers with high stability, biocompatibility, and low immunogenicity (Kim H. I. et al., 2024). Engineered exosomes can deliver ASOs or siRNAs targeting lncRNAs, protecting payloads from degradation and enabling targeted delivery, even potentially crossing the blood-CC barrier, though large-scale production optimization is needed (Zhang et al., 2023). For example, CC cell derived exosomes loaded with siRNA targeting MALAT1, leading to MALAT1 silencing and concomitant inhibition of metastatic colonization (Mashouri et al., 2019). As clinical-grade exosome production advances, this approach may overcome delivery barriers that have hindered nucleic acid therapeutics. Surface functionalization strategies and standardized quality control protocols will be needed to enhance tumor specificity and meet requirements for clinical evaluation.

In summary, lncRNA-targeted therapies like RNAi, ASOs, CRISPR/Cas9, and exosome delivery show immense potential for CC. Overcoming delivery and safety hurdles, coupled with combinatorial strategies, will expedite their clinical translation to improve CC therapeutic efficacy.

## 6 Conclusion and future perspectives

Long non-coding RNAs have emerged as critical regulators in cervical cancer pathogenesis, modulating therapeutic resistance through ceRNA networks, DNA damage repair, epithelial-mesenchymal transition, and key signaling pathways (e.g., PI3K/AKT, Wnt/β-catenin). Their differential expression in patient specimens underscores significant potential as non-invasive biomarkers, while RNAi, ASOs, CRISPR/Cas9 systems, and exosome-mediated delivery offer promising therapeutic avenues.

To accelerate clinical translation, four strategic priorities require focused investigation (Bray et al., 2024). Therapeutic targeting of the ceRNA axis: the predominant resistance mechanism, through ASOs or small molecule inhibitors to re-sensitize resistant tumors, ideally in combination with standard-of-care regimens (Duska et al., 2024). Mechanistic cross-fertilization leveraging insights from other gynecologic malignancies like ovarian and endometrial cancers to expedite understanding, while rigorously addressing cervical cancer-specific contexts including HPV integration dynamics and tumor immune evasion (Olusola et al., 2019). Rigorous clinical validation and current critical bottleneck: large-scale prospective trials are urgently warranted to standardize detection protocols, validate multi-marker liquid biopsy panels, establish safety and efficacy profiles of lncRNA-targeted interventions, and optimize tumor-specific delivery systems such as engineered exosomes and advanced nanoparticles to minimize off-target toxicity (Moody and Laimins, 2010). Personalized medicine integration: capitalizing on lncRNAs as stable, specific biomarkers for early diagnosis and prognostic stratification via minimally invasive detection, deploying them as therapeutic targets to reverse radio- and chemoresistance, and utilizing lncRNA single nucleotide polymorphisms to identify genetically susceptible populations for risk-stratified prevention. Complementing these efforts, future research must prioritize large-scale, multi-center prospective studies with standardized protocols to reduce heterogeneity and enhance evidence reliability, alongside longitudinal monitoring of lncRNA expression dynamics throughout treatment to objectively assess therapeutic response and refine predictive accuracy.

Addressing these challenges through deeper elucidation of lncRNA biological networks will bridge the bench-to-bedside gap, unlocking their full potential as diagnostic biomarkers and therapeutic targets to improve outcomes for cervical cancer patients worldwide.
